# New Books

**Published:** 2006-03

**A Handbook of Globalisation and Environmental Policy**

Frank Wijen, Jan Pieters, eds.

Northampton, MA:Edward Elgar, 2005. 768 pp. ISBN: 1-84376-913-1, $229.50

**Biological and Pharmaceutical Nanomaterials**

Challa S.S.R. Kumar, ed.

Hoboken, NJ:John Wiley & Sons, 2006. 427 pp. ISBN: 3-527-31382-6, $195

**Cancer Bioinformatics—From Therapy Design to Treatment**

Sylvia Nagl, ed.

Hoboken, NJ:John Wiley & Sons, 2006. 288 pp. ISBN: 0-470-86304-8, $130

**Cancer: Cell Structures, Carcinogens and Genomic Instability**

Leon P. Bignold, ed.

New York:Springer, 2006. 375 pp. ISBN: 3-7643-7156-0, $159

**Challenged Earth: An Overview of Humanity’s Stewardship of Earth**

Stephen F. Lincoln

Hackensack, NJ:World Scientific Publishing Co., 2006. 548 pp. ISBN: 1-86094-526-0, $59

**Environmental and Health Risk Assessment and Management**

Paolo F. Ricci

New York:Springer, 2006. 478 pp. ISBN: 1-4020-3775-9, $159

**Environmental Exposure and Health**

M.M. Aral, C.A. Brebbia, M.L. Maslia, T. Sinks

Billerica, MA:WIT Press, 2005. 528 pp. ISBN: 1-84564-029-2, $325

**Environmental Health Risk III**

C.A. Brebbia, V. Popov, D. Fayzieva, eds.

Billerica, MA:WIT Press, 2005. 528 pp. ISBN: 1-84564-026-8, $325

**Environmental Security and Environmental Management: The Role of Risk Assessment [NATO Security through Science Series]**

Benoit Morel, Igor Linkov, eds.

New York:Springer, 2006. 325 pp. ISBN: 1-4020-3891-7, $159

**Genome Exploitation: Data Mining the Genome**

J. Perry Gustafson, ed.

New York:Springer, 2006. 252 pp. ISBN: 0-387-24123-X, $129

**Handbook of Global Environmental Politics**

Peter Dauvergne, ed.

Northampton, MA:Edward Elgar, 2005. 560 pp. ISBN: 1-84376-466-0, $193.50

**Hazardous Chemicals in Products and Processes: Substitution as an Innovative Process**

A. Ahrens, A. Braun, A.V. Gleich, K. Heitmann, L. Lißner

New York:Springer, 2006. 152 pp. ISBN: 3-7908-1642-6, $64.95

**Nature Not Mocked: Places, People and Science**

Peter Day

Hackensack, NJ:World Scientific Publishing Co., 2006. 200 pp. ISBN: 1-86094-576-7, $48

**Policymaking for a Good Society: The Social Fabric Matrix Approach to Policy Analysis and Program Evaluation**

F. Gregory Hayden

New York:Springer, 2006. 251 pp. ISBN: 0-387-29369-8, $84.95

**Principles of Toxicology, 2nd ed.**

Karen Stine, Thomas M. Brown

Boca Raton, FL:CRC Press, 2006. 392 pp. ISBN: 0-8493-2856-X, $79.95

**Proteomics and Protein–Protein Interactions: Biology, Chemistry, Bioinformatics, and Drug Design**

Gabriel Waksman, ed.

New York:Springer, 2006. 324 pp. ISBN: 0-387-24531-6, $139

**The ABCs of Gene Cloning, 2nd ed.**

Dominic W.S. Wong

New York:Springer, 2006. 260 pp. ISBN: 0-387-28663-2, $39.95

**The Implicit Genome**

Lynn Helena Caporale, ed.

New York:Oxford University Press, 2006. 336 pp. ISBN: 0-19-517270-1, $99

**The Nanotech Pioneers: Where Are They Taking Us?**

Steven A. Edwards

Hoboken, NJ:John Wiley & Sons, 2006. 254 pp. ISBN: 3-527-31290-0, $35

**Understanding Industrial Transformation: Views from Different Disciplines**

Xander Olsthoorn, Anna J.Wieczorek, eds.

New York:Springer, 2006. 226 pp. ISBN: 1-4020-3755-4, $109

